# American Dental Convention

**Published:** 1857-10

**Authors:** 


*Treatment of Alveolar Abscess.*

Prof. Harris was the first speaker. He said he did not know that he could say anything new upon the subject, or anything different from what he had stated elsewhere; but a very novel and successful method of treatment was described to him some two or three years ago, by Dr. F. H. Badger, of Nashville, Tenn., which he thought would be interesting to the members of the profession, and he would describe it.

In the treatment of alveolar abscess, it was important that the remedial agent should be brought directly in contact with the inner walls of the sac, and unless this could be done, it was exceedingly difficult to treat it with much success. Impressed with this opinion, Dr. Badger adopted a somewhat novel and ingenious method of procedure. In the first place, he closed the external opening of the cavity leading to the central chamber of the tooth, with gutta percha or gum elastic, perforating this at the most convenient point, and then charging his syringe with the medicated agent he wished to introduce—a solution of nitrate of silver, or an alcoholic solution of tannin and gum benzoin—he injected it into the tooth, and it passed up through the canal, and escaped through the external opening of the gum. In this way the medical agent was brought immediately in contact with the walls of the sac, and with the walls of the fistula opening from it, and which communicated with it. He (Prof. H.) had been completely successful with this method—more so than with any other—but instead of using nitrate of silver, he had used a solution of creosote, as recommended by Dr. Ballard, of New York. There were some cases where so large a portion of the vitality of the tooth had been destroyed, that nothing short of its removal would effect a complete cure. At least he had failed very frequently. After having apparently healed up the ulcerated surfaces, and obliterated the fangs to the very extremities, a recurrence of abscess had taken place perhaps three times in twelve months; although in these cases the amount of matter secreted and discharged through the gums, had always been very small in comparison to the discharge when the root was not completely obliterated.

Some twelve or fifteen years ago, Prof. Harris said, he was requested by a medical gentleman of Baltimore, to visit a patient of his, who discharged three or four times a day a half dozen drops of pus from behind the margin of the palate. The physician had been unable to discover the source of this matter. At first he thought it might come from the maxillary or frontal sinuses; he (Prof. H.) thought it might be secreted by the mucous membrane of the posterior nares. On visiting the patient and becoming satisfied that it came from neither of these places, he passed his finger round under the upper lip, outside of the alveolar border, and immediately over each of the central incisors, a small tubercle about half the size of a pea, was discovered; and perceiving that these teeth had lost their vitality, he had little difficulty in arriving at the conclusion that the pus came from abscesses at their roots, and that it had made for itself a passage through the nasal plate of the superior maxillary bone, and from thence passed back between the periosteum and mucous membrane, perforating by the posterior portion of the velum, and escaped in the manner described. Under the circumstances, he recommended the removal of the teeth, to which the patient readily submitted, and in three days the discharge ceased.

About six months ago, a lady of Baltimore, about fifty years of age, consulted him under similar circumstances, at the request of her attending physician. The first molar on the left side was in a necrosed condition, and from the appearance of the gum, he had every reason to believe that there existed in the alveolus an abscess, and that the matter had come from it. He advised the extraction of the tooth, she submitted to the operation, and in three days was perfectly relieved.

Some two or three years ago, a gentleman called upon him to be relieved from a similar discharge. But a day or two before, his incisors being painful to the touch, and so much decayed that they could not be restored, he had them extracted. From the description he gave of the condition of the teeth, he (Prof. H.) felt convinced that abscesses had formed, and that the matter came from the inside of the sacs, and he assured the gentleman that within three or four days he would be relieved from the difficulty under which he was laboring.

In cases where the matter was discharged through the crown of the tooth, he employed the same treatment, but it would not be necessary to close the orifice with gutta percha, but merely to inject the fluid forcibly, which would carry it into the sac, and in that way reach the ulcerated walls of it.

In answer to an inquiry with regard to the composition of the solution he used, Prof. Harris said, it was about in the proportion of twenty-five drops of creosote, to an ounce of diluted alcohol.

Dr. Roberts said, he hoped to hear a great deal on this subject, for he had treated a great many cases himself, and he supposed other members of the profession had done the same. He believed thousands of teeth were extracted every year, after alveolar abscesses had been formed, which might have been saved by proper treatment, and he considered this a very important point of discussion in the proceedings of the Convention. At least, it was very important to many of their patients that their teeth should be saved, especially front teeth, after abscesses had formed; and his success in the treatment of such cases induced him to state the method he pursued. He injected nitrate of silver into the root, and continued the injections until the matter ceased to flow from that point. Then he introduced with a small broach, floss silk saturated with creosote, as far as possible clear into the alveolar process. At first, he let it remain one, two or three days, so that the matter should not form and dam it up, and cause pain. When it was removed, it threw off a very bad odor. This process was to be repeated from day to day, and they would find it would take perhaps months to cure the disease. After the tooth was entirely cured, they should put their gold clear to the very point of the root, and build from that.

Dr. Roberts was asked what he would do if an abscess should form after the tooth was filled, and replied that it would never be so large as before. He said further, in answer to a question, that he did not know the exact proportion of nitrate of silver he had used; it was such as was used for the treatment of gums, and could be obtained of the apothecaries.

Dr. Field, of Waltham, said his experience in this branch of practice had not been very extensive, but where there had not been a fissure open enough to inject the fluid, he had found that by drilling through the alveolar bone, just above the roots of the tooth, he could reach the disease. In such cases, after he had cleared out the cavity, and filled under the root clear to the very point, he then opened the abscess with a little broach, and let the matter out. It seemed to him, that when the sac was full of fluid, by simply injecting through the root, they could force fluid enough to mix with that of the tooth, to answer their purpose. He had done it in every conceivable way; he did not use floss silk, but prepared flax, which was very fine indeed.

Dr. Roberts thought the difficulty with flax would be, the liability that a piece would be left in the cavity.

Dr. Field did not think there was much danger of this.

Dr. Mowe, of N. H., said he took considerable interest in this subject, and gave an account of an operation he performed on an incisor, which had been filled, and apparently well filled. He took a small drill and opened into the dental canal, which he enlarged to the end of the fang, as near as he could judge, and was able to see into it. Then he applied pure creosote, on a bit of cotton, to the end of the cavity, and filled it up with cotton. The patient came again in three days, and the sac formed by the abscess had disappeared. He made another application, and in ten days it was all well, and he filled the tooth, and it had never given her any trouble since. This was three years ago. He had treated another case, where an abscess had gathered on each of the four superior central incisors, and the alveolar process was much affected, in the same way, curing one tooth at a time. All he used was creosote, applied as he had described.

Dr. Roberts said that he should think ten days or even fifteen, a very short time to allow for the operation.

Dr. Mowe stated that he steeped the gold in creosote, before putting it into the tooth.

Dr. Dwinelle said the treatment of alveolar abscess was much better understood now than formerly. An abscess of this kind was evidence of the presence of a foreign substance—in all probability, a dead nerve—which nature was trying to dispose of, just as she did when a grain of dust got into the eye. Common sense said the first thing to be done was to remove the foreign substance; consequently, he recommended the removal of the nerve. The bone of the tooth was not solid; it was full of little cavities, which were full of a foreign substance, the residuum of the dead nerve, which must be absorbed. The best method was to insert the oil of creosote upon floss silk; let it remain twenty-four hours; renew it and insert again, charged with creosote, and so on; then fill the nerve cavity. Still the abscess was there, and had got to be disposed of. Plunge in the lancet; then take a sharp instrument, which answered the double purpose of a probe and an excavator, and plunge it down to the very extremity, and examine and ascertain if there is any foreign substance there. They would often find a deposit of salivary calculus, or something of that sort; such as had been found in the brains, the lungs, even the larynx and other parts of the system. If any was found, it should be all scraped off. Here another principle of the animal economy came in to insure their success, and that was, the scraping of the bone excited inflammation and this caused nature to throw out a healing fluid, which came to the assistance of the operator. They had then opened the way for the discharge. After the discharge, a drop of creosote was again to be introduced, and the operation was completed.

Dr. Miller, of Worcestor, remarked that, although the subject was of great importance, he should say but little at this time. He had pursued the methods of cleansing the fangs, and injecting various solutions into the sacks, as described by previous speakers and in addition, had used the lancet freely—cutting deep; and into the incisors, with suitable instruments, introduced a granule of crystallized nitrate of silver, one-fourth to half the size of a grain of wheat. He had seen good results from this practice, and would strongly recommend it to the profession. One case, he would mention, however, embracing the superior central incisors, which had entirely baffled his skill. At the time the patient applied to him, the ulcerations had existed a long time, producing an unnatural fullness of the mouth. The openings to the sacs were free—the nerve canal being much enlarged. In cleansing the fangs with a little cotton twirled around a small broach, and dipped in a solution of whatever was used at the time, the patient would frequently experience sharp pain like that produced from cutting inflamed dentine.

The question was here asked if the pain were not caused by pressing air into the sac.

Dr. M. replied that it was not, as he had thought of that, and used an instrument much smaller than the nerve canal. Careful examinations, at various times, proved the existence of morbid sensibility of the parietes of the dental canal, not, however, equally disseminated over the whole surface. After treating the case from four to six months, without the slightest improvement, it was abandoned.

Dr. Wetherbee, of Boston, described a case which had been under his care, where there was an abscess on each of three incisor teeth and the cuspidati, which had been filled. The case was a very difficult one, from the fact that there was so many decayed nerves and so great an amount of inflammation. He removed the fillings, and out gushed any quantity of offensive matter. He syringed out the cavities faithfully, and also prepared an instrument for scraping the surface of the nerve cavity, as far as he could. This operation he performed on all four of the teeth. He then injected cologne water, for some length of time, and afterwards filled the cavity with cotton, and requested his patient to let it remain several days. At the expiration of this time, she called again, and he found the emission from the gums had somewhat subsided. He finally plugged the teeth without any further introduction of liquids to the nerve cavities. After plugging the teeth thoroughly, he took a sharp excavator, and commenced upon the alveolar process, scarifying the gum, and scraping the alveolar process for some distance round the centre of the opening of the abscess, until he felt confident he had broken up every vestige of decomposition, occasioned by the abscess upon the alveolar process. He then dismissed the case. The lady was much pleased with the operation, and there had been no return, so far as he knew, of the disease. He believed, with Dr. Dwinelle, that, by thoroughly scraping and removing every remnant of the disease, and by injections, the difficulty, in ninety-nine cases out of a hundred, could be removed.

Dr. Forbes, of St. Louis, gave an account of a very interesting and peculiar case which had come under his observation, with regard to which, he desired the benefit of their experience and knowledge. The case was that of a lady whose upper central incisor, on the right side, was attacked so virulently, that the dentist she consulted thought proper to extract it. The disease spread, attacking one tooth after another, which were successively extracted, but without curing the disease. The lady thought she had necrosis, or a disease of the jaw. She had been treated with chloride of potassium, zinc and creosote. In his absence, he had prescribed constitutional treatment. It was so interesting a case, that he had called in quite a number of the dentists of St. Louis to consult upon it, and he would like to have the advice of his brethren. [Dr. F. gave a minute account of the present condition of the lady's mouth, but his remarks, from the position he occupied, and the low tone in which he spoke, were, in great part, inaudible to the reporter. The same difficulty was realized with regard to several of the other speakers.]

In answer to inquiries, Dr. F. stated that the patient was a lady, of healthy constitution and simple habits, and twenty-five years of age; that the emission comprised two to three pustules of pus during the day; that there was no dead bone discoverable by the probe; that he could not probe more than a line, or a line and a half; and that it was not an hereditary disease.

Dr. Straw, of Bangor, recommended an abstemious diet, as likely to do more than anything else.

Dr. Locke, of Nashua, said his experience did not confirm the use of the powerful remedies which had been referred to. He had not been successful in the use of creosote; he had found that the tooth became a source of irritation in the socket, and he had ultimately lost it. He had also tried an injection of nitrate of silver, and in two or three cases, had benefited the patient by its use; but he did not think he had benefited any case by the use of creosote, and he feared if it was used as freely as had been described, the result would be the destruction of a great many teeth, which might be useful if they simply adopted the "let alone" principle. He had used tincture of nut-gall and tincture of tannin, and he thought he had produced better results from their use than by anything else. He had cured several cases with them, and he thought them the safest and most useful remedies for the disease. If the case was not a very troublesome one, it was his experience that it was not best to meddle with it. He was pleased with the suggestion of Dr. Miller, and thought it might be useful in a good many cases.

Dr. Allport suggested the practice of opening into the abscess, and the application of creosote on a pledget of flax cotton, in immediate contact with the apex of the fang—a vigorous application so as to break up the abscess.

The Convention then adjourned to meet on Thursday morning, at 9 o'clock.

*Third Day.—Forenoon Session.*

The Convention was called to order shortly after 9 o'clock, by the President.

The record of Wednesday's proceedings was read by the Secretary, and appproved.

The Chair announced the first business in order, to be the fixing of the time and place for holding the next Convention.

Dr. Hawes, of Providence, moved that when the Convention adjourn, it be to meet at West Point, on the first Tuesday in August, 1858.

Dr. Perine moved to amend, by substituting Cincinnati for West Point.

After a brief debate, the amendment was adopted, and the resolution as amended, adopted.

Dr. Goldey, of New York State, moved a reconsideration of the vote. He wished the Convention to meet at Buffalo.

The motion to reconsider was put, and lost.

On motion of Dr. Bonsall, a committee of two was appointed by the chair to audit the Treasurer's account, as follows :—Drs. Bonsall, of Cincinnati, and Perine, of New York.

On motion of Dr. Allport, the paper by Dr. Westcott, on "Cheoplasty," presented by him yesterday was accepted, and a copy ordered to be furnished to the Dental Journals for publication, if they desired it.

Dr. Johnson then moved a reconsideration of this vote, for the purpose of referring the paper to a committee, and the reconsideration was carried.

Dr. Johnson moved that a committee of three be appointed to examine the paper, and report upon it.

Dr. Spaulding, of St. Louis, offered an amendment, giving the committee full discretionary power in the matter, which amendment was accepted by Dr. Johnson, and the motion adopted.

On motion of Dr. Allport, it was voted that the committee consist of the editors of the three Dental Journals.

Dr. Townsend, from the committee appointed last year, to consider the expediency of the establishment of a uniform system of prices, submitted a report, which was listened to with close attention, and frequently applauded.

As the resolution appended to the report contains the substance of the whole matter, we give it only.

*Resolved*, That in the judgment of this Convention, the formation of county, town and neighborhood societies of Dentists, is among the best of all methods for the government of their social relations, their mutual improvement, the harmony, dignity and elevation of the profession, and that to such distinct organizations, properly belongs the regulation of fee-bills, the repression of quackery, and whatsoever in the practice, conduct, qualification and character, of individual practitioners, concerns the general well being of the profession.

It was voted, on motion of Dr. Spaulding, of St. Louis, to accept and adopt the report and resolution.

Dr. Miller, of Worcester, (a member of the Business Committee,) then addressed the Convention briefly on the subject of the preparation of business. He concluded his remarks by offering the following resolution, which was adopted:

*Resolved*, That a committee of five be appointed by the chair to prepare and arrange the order of business, and subjects for discussion, at our next annual meeting, and that said committee announce the result of its deliberations to the Dental profession, either by circulars addressed to members individually, or through the medium of the several Dental periodicals, as early as January, 1858.

The committee appointed to examine the Treasurer's accounts, reported that they had attended to that duty, and found them correct, and that there was a balance in the treasury of $40 56. Report accepted.

### *Miscellaneous Business.*

Dr. Sylvester, of Lyons, N. Y., at the request of a member of the Convention, exhibited a plate, which he said was made as a temporary plate of 18 or 20 carat gold, with alloys of copper, silver and platina. The plate was worn about a year, and when removed for the purpose of constructing a permanent plate, it was found that its palatial surface had become coated with what appeared to be quicksilver, while the lingual surface was in its normal condition.

Dr. Dalrymple, of New York, said that some eight years ago, he made a set of teeth, and two weeks after they were put into the patient's mouth, they were returned to him, owing to the plate being, as it was said, of impure gold. He was unable to account for it, and consulted some twenty dentists, neither of whom could give any explanation. But Dr. Reynolds, now of Waterbury, who was then a student in his office, told him that if he boiled a plate in tin, it would have just that same appearance.

Dr. Sylvester—Then the appearance would be on both sides.

In answer to questions, Dr. S. said the plate was pickled in a porcelain dish; that the male die was of zinc, and the female of lead.

Dr. Branch, of Illinois, mentioned a case where it was complained that he put bad gold into a plate. On examining it, he found it spotted over with what he considered oxyd of mercury. The gentleman said it could not be so unless he (Dr. B.) put it there. On looking into the matter very closely, he finally ascertained that the appearance was caused by a preparation of chalk and mercury, which the patient, a physician, had had on his fingers when handling the plate. Though the preparation was brushed off from his hands, there yet remained enough to discolor the plate; and, perhaps, the one exhibited was effected in the same way.

Dr. Sylvester, in reply to this suggestion, said that one side was entirely covered and the other entirely free.

Dr. Roberts stated that he had removed a clasp from the mouth of one of his patients, which presented a similar appearance. He found that it was caused by quicksilver that he had used and that had got on his hand at the time of casting it.

Dr. Spaulding, of Dedham, suggested that such effects demonstrated the importance of investigating and understanding the physiological condition of the system. If it was a fact, that a plate inserted into the mouth could extract poison from the system, it was an important fact to be understood by every medical man. If he understood the matter, the second plate was not affected in the same way. It was a matter of importance and interest to their profession, that they should understand well what deposits could be made from the fluids of the mouth, or from anything they could do in the mouth, by the insertion of plates or fillings. If they found that the system was surcharged with mercury, so that it made a deposit upon the plates, means should be used either to relieve the system of this amount of mercury, or correct the secretions of the mouth, so that their plates should not be affected by them.

Dr. Goldey, of Oswego, said that three or four years ago he inserted a plate, on which, at about the end of a year, he found quite a heavy deposit of mercury. He boiled the plate in acid, and removed the deposit without difficulty; he put it in again, and in about a year, found about the same quantity deposited upon it. The patient was not conscious that she had ever taken any mercury, but it was quite evident that she had. She was uncommonly healthy. He had boiled the plate twice, but there was even now a slight coating upon it.

Dr. Dwinelle was then called upon, and addressed the Convention. He said, perhaps, there was no department of their art which made it approximate so much to a divine art as that department which had for its object the regulation of the teeth, and the consequent regulation of the general deformities of the mouth.

One great difficulty in the way of correcting irregularities and deformities of the mouth had been the inability to make fixtures of sufficient strength to answer their purpose, while they were small enough to be practicable. A few years ago, having this in view, he undertook the construction of a new instrument for that purpose. He found an old case of surgical instruments that came, originally, from London, and on the hilt of each instrument was screwed or consolidated a piece of zinc; and he found, on reference to his library, that this was often resorted to to prevent valuable instruments from rusting. He thought of making his instrument of steel, small enough to be practicable, and applying the zinc to prevent rust. His mind at once reverted to the simple screw and nut, or what is familiarly known as the jack-screw. He went to work and made some of these, not over an inch or an inch and a quarter long. This instrument was susceptible of every variety of modification, and could be used at any angle. It would not rust in the mouth enough to injure it, but the zinc ends required renewing occasionally, on account of the waste by galvanic action. He thus had an instrument capable of spreading the mouth almost entirely apart, at the same time that it was not in the way, as other fixtures were. His patients preferred this method of operating to any other which he had adopted. One reason was, that the pressure was firm and altogether in one direction; it did not admit of a vacillating motion, backwards and forwards, which was conducive to undue inflammation; the constant pressure, too, operated to close the blood vessels, so as to forbid any excessive inflammation. [Dr. D. explained, by diagrams, his method of using the instrument.]

Dr. Clark, of New York, inquired if Dr. D. did not find that the life of the teeth were destroyed?

Dr. D. replied that they were not. There was sufficient circulation on the other side. With this instrument he had forced the central incisor, in a patient 33 years of age, from a position where it was a serious interference with the articulation, into its proper position in the arch, in five weeks, without any trouble whatever. He had had several cases where the patient was 18, 20, or 25 years old, but he thought this case of 33 years was remarkable. He felt confident that he could succeed in all cases, even at that age, unless the patient was of a scrofulous habit, and then he could modify the difficulty. He fixed the instrument in its proper position by attaching plates to the teeth, in which he set the points.

Dr. D. then described a very interesting and difficult case which had been under the care of Dr. Dalrymple, of New York City, and which Dr. Perine had previously alluded to. The patient was a young lady, fourteen years of age. At birth she had a hair lip, which deformity had been corrected as far as possible; but as her teeth developed, she had no frontal incisors; originally, an elemental or supernumerary tooth came in and occupied the place of all the frontal incisors. The articulation arranged itself in such a manner that the upper were continually embraced by the lower teeth; ultimately, they must have been brought almost in contact. The arch was exceedingly narrow. The two superior cuspid teeth would have been brought entirely together in five years, and any one would see the consequences; the palatine arch must have been closed up, and all the unhappy consequences which would naturally follow would be the result. Dr. Dalrymple, after working a year, succeeded in separating the cuspid teeth so as to get sufficient space for the four frontal teeth; he supplied the lost incisors, and almost entirely corrected the deformity, changing the expression from an unpleasant and almost hideous one to a very pleasant and agreeable one.

Dr. D. exhibited a cast of the mouth taken before the operation, which was certainly sufficiently deformed, and also the cast taken when the work was finished, which looked as if nature had done her best to give the lady a perfect dentition. In conclusion, he expressed the hope that Dr. D. would himself explain the case, for he felt that he had not done him justice. He was entitled to a great deal of credit for the operation, and he, (Dr. Dwinelle,) honored him for it. He knew the difficulties in this comparatively untrodden field; for, with all respect to the past, it was almost entirely a new field.

Dr. Dunning asked Dr. Dwinelle if he found many cases in which the jack-screws were available? He did not see how, in many cases, they could be applied in pushing forward in the right direction.

Dr. D. replied, that it was often necessary to construct an opposing surface, artificially fashioned.

Dr. Roberts inquired what could be done with very young patients, where it was almost impossible to attach any fastenings to the teeth?

Dr. D. said, in that case he made them, and explained on the black-board how he did it. He said he often had to break up the articulation altogether. The patient masticated with the fixture in his mouth. He had moved a tooth half an inch, by actual measurement, in this way. He straightened out a lady's teeth, which were "confusion worse confounded," in five month's time; in any other way it would have taken a year. Oftentimes the right direction could be given to children's teeth by seeing them two or three times; for the adage, "just as the twig is bent the tree's inclined," never applied more forcibly than in their profession. Simply by the use of a rubber band, or a piece of pine wood, he had corrected the worse kind of deformities. In the case of a child, the teeth often had to be tied in their place after they were got there. Dr. D. said, he did not think it safe to fill teeth before they became fully developed, as there was danger of inflammation; still, it was necessary to do it sometimes, but it should be done with great care.

Dr. Dalrymple, of New York, then explained the operation to which Dr. Dwinelle had referred. His description was substantially the same, but more minute in the details. He said he saw the patient once a week, on an average, for fifteen months, and had every conceivable difficulty to contend against. He consulted Dr. Dwinelle several times, and received many valuable suggestions from him. One very gratifying change, caused by the operation, was the alteration in the appearance of the upper lip. The work was performed with wedges, and he found, at one time, that an inflammation had been excited; but, on reflection, he came to the conclusion that was just what he wanted, as the result proved, for the inflammation he had excited, caused the cartilage of the nose, (which had been drawn down by the operation for the hair-lip,) to relax and allow the lip to take its natural shape; and the result of the whole was that the patient was now a pretty good looking girl.

Dr. Allport exhibited some models of a case operated upon by Dr. Elisha Tucker, where the upper teeth lapped over a great deal, which had been treated very successfully, and also described an operation of his own, which he said was half as bad again as that. His motto always was "Try!" and he never allowed a case to come to him, however difficult, without at least making an attempt to remedy the evil. The ordinary way of operating in the case to which he referred, would have been to supply elastic ligatures; but the trouble would have been, that the ligatures would slip up and irritate the gums, and he concluded he would not try them. He attached plates to his molar teeth, and by sundry ingenious mechanical contrivances, (which he illustrated on the black board,) effected his object. Dr. A. expressed his belief that the principle stated by Dr. Dwinelle was the only correct principle of regulating teeth; the tooth was drawn to its place, and the blood vessels had no chance to become injected, consequently, there was no inflammation.

At the request of a member Dr. A. then gave an account of a case of fracture which had come under his hands. It was that of a gentleman who was thrown from a hack, and kicked by the horse. The blow was upon the left side of the face, and it broke through the whole jaw, so that when the case came to him, the molar teeth occupied the centre of the mouth. The surgeons of the hospital concluded they would rather not undertake it, and finally asked him to see the patient and decide whether he could do anything. The trouble was to get the jaw back to its place and hold it there. He could think of no way without a plate, and the question arose how to get a plate. He found it impossible to take an impression of the whole mouth in the ordinary way, and then tried to take half the mouth; but when he undertook to take off the wax, it tore all to pieces. Finally, after taking the impression, he filled a large syringe with ice water, with which he hardened the wax in the mouth, and then removed it with without trouble, and thus got an impression of half the mouth. The other half he did not *take*, but *made*, by the half he had, and struck up a plate which was as the mouth would have been if it had been perfect. He took the plate to the hospital, and, with the assistance of the surgeon, removed considerable bone and some flesh that hung loose, and then pressed the plate into the mouth, and got each tooth into its proper place. He then bound the jaw firmly, and left him. The operation was perfectly successful.

Dr. Dwinelle stated, that some three years ago Dr. Miles told him that he had often regulated cases of deformed teeth in children, when the gums were in a mobile state, on the instant. Having cut about the tooth, he got the bearings on one side and the other; he would take a large forcep and press the tooth back at once. He mentioned this to show how accommodating the animal economy was.

Dr. Clark, of New York, said perhaps some would like to have a simple means of correcting cases that occurred in every day practice. In cases where the tooth turned round, from one-quarter to one-half, he fastened a gold spring round the central incisor, instead of making a plate, as was common.

Dr. J. G. Coates, of Virginia, explained and illustrated, in a very happy manner, his method of operating on deformed mouths. After fitting the plate, he sawed it in two in the centre, and moved the teeth by means of screws attached to the sides. In seven weeks, in the case he described, he had the teeth in perfect form.

Dr. Sanborn, of Andover, remarked that a good deal had been said about preserving the teeth of the young, and perhaps it would not be amiss to mention a very simple method of securing the teeth of old people, which teeth had become loose. He took a small platina wire, drawn for the purpose, and wove it round and between the teeth, then cut the wire off, turned it down, and then the teeth were as firm as ever. He had used this method on the teeth of several old ladies and gentlemen, three, four and five years ago, and they were still firm and doing good service. "In union there is strength."

Dr. Forbes, of St. Louis, exhibited a very simple invention, made by Dr. J. H. Fairbanks, of St. Louis, for cooling wax in the mouth. It seemed admirably adapted to its purpose.

Dr. Roberts explained a method he had adopted to enable him to use the inclined plane to regulate the teeth of children, where it was difficult to get anything to rest solid on the lower jaw. He took an impression of the teeth in gutta percha, which gave a more perfect representation of the points of the lower teeth than was obtained in any other way, and then made a plate of platina.

Prof. Harris, of Baltimore, then stated that, at the request of the Boston Committee, he would move that the Convention adjourn at half past one, and proceed at once to the boat for the excursion in the harbor.

While up, Prof. Harris continued, I must take occasion to express my deep regret at not being able to partake of the hospitalities tendered by our Boston brethren, having just received a letter which makes it necessary for me to return home at once. I regret this the more, knowing the character of our Boston friends, and that they have acquired somewhat of a celebrity, especially for the excellency of their tea parties, (loud applause,) having given one more than eighty years ago, (applause,) and provided a cup so large and so fragrant, that the odor thereof diffused itself through the atmosphere of our whole country, (renewed applause,) inspiring the people of our then infant colonies with such an ardent love of liberty, that they all came up as one man, and broke the chain which had held them for years and years in bondage. (Enthusiastic cheers.) It is, therefore, a source of deep regret to me that I cannot be with them and participate in the festivities of the occasion.

The motion was unanimously adopted.

Prof. Harris then said, at the request of Dr. Allport, he would say a few words by way of caution, in regard to changing the position of a tooth too rapidly. Inflammation of the periosteum and pulp might be excited, which would terminate in the disorganization of this highly important portion of the tooth. Not long ago, a most interesting and highly accomplished young lady was brought to him by her father, with the view of ascertaining if something could not be done to restore the lost color of a tooth, which had been but recently brought into the dental arch. He at once perceived that the pulp was in a state of disorganization; that the tooth had lost all that portion of its vitality which it derived from the lining membrane; and, on inquiry, he found that the position of the tooth had been changed more than a quarter of an inch, in some five or six days, by the pressure which had been applied to it, for the purpose of bringing it forward to its proper position. It was done by a band of india rubber, assisted by the action of an inclined plane. That this would always occur was by no means probable; on the contrary, he knew it would not; but it was liable to occur in individual cases, where the system was more than ordinarily susceptible to the action of irritants; and in such cases the teeth should be moved much more slowly than when no preternatural excitability of body existed. He wished to caution gentlemen in this matter. He had known the same thing to occur from the action of an inclined plane, when made to act too promptly upon a deviating tooth.

Dr. Forbes, of St. Louis, exhibited and explained an impression plate, the invention of Dr. Fairbanks, of that city, which attracted a good deal of attention.

Dr. Branch, of Illinois, exhibited his invention for producing local anæsthesia. He said some complaint had been made with regard to its operation; that he thought that was occasioned by the operator not understanding the proper method of applying it. He had found it a perfect substitute for chloroform, in those cases where he felt a desire to use chloroform. It was harmless in all cases where chloroform was most objectionable. He did not mean to say that it could be profitably used in all cases, but that it was of so much benefit that no dentist could afford to do without it. It was now in use in general surgery as a remedial and anæsthetic agent.

Dr. Dwinelle called on Dr. Wetherbee, of Boston, to state his method of preparing the mouth for the reception of an entire dentition.

Dr. Wetherbee said that he ever regarded the preparation of the mouth for the reception of artificial teeth as of great importance. In a very few words he would describe the mode he adopted some nine years ago, and which he pursued at the present time. Dr. W. then described his process, the peculiarity of which was, that in preparing his temporary plate, he allowed for the probable shrinkage of the gums. In some cases he attached springs to be worn until the plate adhered. The result was, that at the end of the year, the plate set as well as the permanent plate would, the mouth was smooth, and when he introduced his permanent plate, it set very firmly indeed.

Dr. Austin, of Manchester, N. H., described a new invention for the application of cold air, to secure local anæsthesia. It consists of two or three cans, placed one within the other, the inner can being filled with ice and salt, and a pipe conveying the air to the mouth, which was forced up by an ordinary pair of bellows, worked by the foot, or in any other way.

Dr. Branch asked why it was called an invention for securing *local* anæsthesia.

Dr. Austin replied, that as soon as the air ceased to be local the other air mingled with it and moderated its temperature.

On motion of Dr. Rogers, the discussion of miscellaneous subjects was suspended, to hear reports from committees.

Dr. Dwinelle, from the Committee on Printing, appointed last year, presented a report which was accepted.

The same gentleman reported from the committee to whom was referred the communication on plastic gold, received a few days since, from Dr. Weiber, of Paris, that the gold was about the same as manufactured in this country some six years ago, and rejected as not adapted to the wants of the profession. They recommend the Convention to confer an honorary degree upon Dr. Weiber, and to send him samples of gold manufactured in this country at the present time.

The report was accepted and adopted.

Dr. Dwinelle now made some remarks *strongly* condemnatory of the course pursued by a Mr. Gilbert in patenting an improvement known by the name of the "Central Cavity Plate," and which he said had been used by the profession long prior to the date of said patent, also stating that one of their members, Dr. Potter, of Norwich, had been sued by said Gilbert for a violation of the patent, and that he thought an expression from the Convention, of sympathy and aid to Dr. Potter, was proper and just.

After some remarks from other gentlemen, fully endorsing the views advanced by Dr. D., resolutions expressing sympathy and offering aid to Dr. Potter were offered and carried with great unanimity and applause, but which, on subsequent action, was re-considered and laid upon the table, in consequence of the opinion prevailing that their action would lessen the weight of their evidence, as parties interested, in case any should be called upon as witnesses in the suit now pending.

Prof. Taft, in behalf of the committee to whom was referred the essay by Dr. Westcott, stated that in glancing over it hastily, they noticed that it was upon a subject which had not come before the Convention, and probably would not, and as it was predicated on the supposition that the subject would be introduced, that under these circumstances, they did not deem it important that the paper should be read to the meeting. The report was accepted.

Dr. Locke, of Nashua, said he had prepared some resolutions which he desired to present to the Convention, and which, if there was not time to act upon them at the present session, he hoped they would be permitted to appear among its proceedings:

*Whereas*, We deplore as much as the public can, the physical degeneracy of the human race in so-called civilized society, as manifest to the physician, in the enfeebled constitutions and the diminished stature, as to the dentists, in the narrowly contracted maxillaries, and in the imperfectly developed dentures, which renders them the most irregular and defective in parts of speech known in the English language:—therefore,

*Resolved*, That in the opinion of this Convention the causes of imperfectly developed and defective teeth arise, first, from the violation of the physiological laws in the formation of the marriage union; second, from the want of a sufficient supply in our diet, of the chemical elements which are needed to form *bone;* third, by too free use of acids in our food and drink; fourth, mainly from acetic acid, formed by fermentation in the chemical laboratory of the mouth and stomach from sugar and saccharine matter in all its forms, and from most articles of food, when more is taken than is digested.

*Resolved*, That the remedies we now know of are, first, cleanliness of the teeth and mouth; and second, a temperate use of the coarser and more substantial articles of food.

On motion of Dr. Spaulding, the resolutions were laid on the table, to become a part of the proceedings of the Convention.

On motion of Dr. Straw, of Bangor, it was voted that when the Convention adjourn, it be until to-morrow morning, at 9 o'clock.

[The Convention then, at half-past one, adjourned, and proceeded in a body to the steamboat which had been procured by the dentists of Boston and vicinity for the purpose of an excursion down the bay. On the boat was found a band of music, and a very liberal supply of refreshments, which was partaken of with keen appetites. During the trip down many speeches were made, and we are compelled to add, many stomachs relieved in consequence of—not the speeches or the clam chowder—but from a very peculiarly undulatory movement of the water. Notwithstanding these interruptions, the boat reached "Nahant," (where, it will be remembered, the "sea serpent" is occasionally seen,) when, after a sojourn of some two hours, the party again embarked and made their way slowly up the bay, touching at Hull, where a very *spirited* entertainment was given by one of the gentlemen of the reception committee, Dr. E. G. Tucker, of Boston. The boat was again manned, and on the way up to Boston, speeches were again in vogue, and "all the world and the rest of mankind" were called out. At 10 o'clock P. M., Boston was reached, and the festivities terminated.]

### *Fourth Day—Morning Session.*

The Convention was called to order at a quarter past 9. The Secretary being absent, Dr. Forbes, of St. Louis, was appointed Secretary *pro tem.*

On motion of Dr. Taft, the reading of the minutes was dispensed with. The same gentleman then moved a reconsideration of the vote, whereby the Convention voted to lay the consideration of miscellaneous business on the table, and the motion was carried.

### *Miscellaneous Business.*

Dr. Simonds, of Boston, addressed the Convention on the subject of extracting teeth. He said he had some experience in this branch of practice, and for the last six years, he had made it a rule to extract as many as it was necessary to take out, at one sitting. He had generally found that when he succeeded in clearing the mouth from carious teeth at one time, the patient got along better, and did not seem so much debilitated, as when the operations have continued from day to day until the mouth was cleared. He had extracted the full dentition, thirty-two teeth in one instance, at one time, and the patient got along without being sick. Frequently he extracted twenty or more at one sitting, and never had had any bad consequences follow these operations. This was an operation, he conceived, which required a great deal more skill and judgment than many were willing to admit. He thought it unwise to attempt to deceive the patient; if he wanted to see the instruments, let him do so; make the operation appear as bad as possible to his mind, and then when it was over, he would be likely to say it was not half so bad as he expected.

Dr. Taft called upon Dr. Blakesley to address the Convention.

Dr. B. said, it seemed to him, that there was a definite way of getting at the matter of diseased teeth, and the remedy; and the thought had occurred to him, that it would be well for the Convention to institute a fund and employ one or more competent persons, whose time should be occupied in the analysis of the various grains and vegetables in their natural state, and after they were cooked, and see how the system is affected by them. He was sorry to say he did not know what a grain of wheat or corn, or a potato contained; he wished he did; but he had no time to analyze them for himself, and wanted all this matter put into such a condensed shape, that in a few minutes he could ascertain all he wished to know. He was willing to be taxed from ten to fifty dollars a year to promote an object of this kind. (Applause.)

Dr. McElroy, of New York, recommended Dr. Johnson's "Chemistry of Common Life," as containing all the information desired on this subject.

Dr. Taft, of Cincinnati, said this was a subject in which he had been very much interested, but it was only by accident that he ascertained that the thoughts of Dr. Blakesley and himself had been running in the same channel upon it. Every one could see the propriety of this, and the beneficial results that would accrue to the whole profession if Dr. B's suggestion was carried out. If he understood the idea, it was, that their society should raise a fund to sustain an experimenter for the benefit of dental science. They all had very many things coming under their observation, about which they were uncertain, and they resolved at some time or other, to institute experiments, and find out the truth ; but that time never came. Take, for instance, decayed teeth. Did not the discussions of the last three days show that there was no fixed opinion with regard to the cause ? Yet it could be ascertained ; there was no doubt about it. The nature and cause of decay could be brought out, and based upon facts, so that they could all understand them, if some one would take hold of the subject, and work away at it until he had attained the truth of the matter. How many other things there were, about which there was the same uncertainty ! If this fund was raised, when such cases occurred, they could refer them to their scientific experimenter, and ask him to investigate them, and find out what was in them. Every one would see how a hundred good objects would be subserved by this.

With this object in view, Dr. Taft said he had drawn up a series of resolutions, which he thought covered Dr. Blakesley's idea. He read the first, as follows :

*Resolved*, That this Convention establish a fund for the promotion of dental science, with especial reference to the employment of some competent person or persons, to conduct experiments—physiological, pathological, chemical and hygienic—as connected with dental science.

Dr. Taft said, that by the appointment of this agent, they would all obtain more than any one, even under the most favorable circumstances could. The position he occupied would give him the command of resources, which no individual under other circumstances could command, for he could call in the aid of eminent chemists and other scientific men, all over this country and in Europe, who would be willing to lay everything they had at his service. Then the results could be published through whatever medium they desired.

Dr. Blakesley wanted this Convention to have the merit of originating this movement, carry it on in their own way, and publish the results in their own way.

Dr. Branch desired to second the motion, and back up with all the power he had what Dr. Taft had said on the subject. He presumed he had one hundred things in connection with his profession which he desired to investigate, but he could never find time to do it; and although he found these things treated of in books in a general way, he did not have them treated of in reference to his profession. They would save themselves and patients any amount of imposition, if this plan was carried out. They needed to have some one in their employ, whose decision in such matters would be authority—upon whose opinion, with regard to the action of certain kinds of nutriment upon the constitution and the teeth and kindred subjects, they could rely. He believed this was just what they wanted, and, as has been said on another and greater occasion, "he gave his hand and his heart to this vote."

Dr. Lord, of New York, remarked, that with all due respect to the gentleman who had introduced this resolution, and to those who had advocated it, he could not see the propriety of it. If this society had been instituted twenty-five or thirty years ago, then such a course would have been much more consistent and proper, but now the field had been gone over, the science of chemistry had been very fully developed with reference to every branch of art.

Dr. Buckingham, of Philadelphia, opposed the resolution. He wanted one society in the country, where every man could come in and speak his thoughts, and when he went away, profit by what he had learned. He was afraid the plan proposed, if carried out, would restrict the freedom of the Convention.

Dr. Straw, of Bangor, suggested that it was easier to pass resolutions than to raise funds. He should like to know how they could raise a fund. They had no organization by which they could assess their members, and it seemed to him, that it was not proper to pass the resolution until they knew they could raise a fund in some way or other.

Dr. Kendrick, of Mississippi, favored the resolution, as a matter in which they could all take pride. He had often been gratified by hearing persons say, when speaking of dentistry, "why, really, your profession is getting to be a scientific one." He did not consider it necessary that they should be an organized body in order to secure this object. There were thousands of dentists scattered through the country, who would be glad to contribute something to this end. It had been suggested, that men might come there and present their views, but it must be remembered that some of their ablest men were the poorest—their mind was their only capital—and they could not afford to suspend their business, and come and think for them. If they did so, it was no more than right that they should be paid for it. Dr. K. suggested that a circular should be sent to every dentist in the country, stating what they wanted to do, and requesting him to enclose a gold dollar or more, in a letter to the Secretary, in furtherance of the object.

Dr. Taft said there would be no difficulty in raising funds, if they decided to do it. They could easily find ten men who would give from ten to one hundred dollars a year. He hoped they would begin now, and they would have at the end of a year, one hundred results—for which he was willing to pay one hundred dollars—by which they could all profit. He was astonished that any man should think that the science of chemistry was now perfected, and had reached its culminating point, especially, when they came to bring chemistry to bear upon their profession, and saw how little was known upon the subject, and began to open their eyes to the fact that they knew nothing at all. (Loud applause.) If any man knew all about it, let him tell it.

Dr. Dalrymple stated that Dr. Westcott, of Syracuse, one of their own members, was now preparing a work to show the different effects of the different kinds of food and condiments upon the teeth, and in the course of time they would see that work published to the world. It was to be in a condensed form, that every man could have it in his library.

Dr. Allport spoke in favor of acting upon the matter now. If they suffered it to lie over until next year, they would lose the benefit of one year's experiments. That was no small item; for every determined fact in their profession, not only accrued to their advantage, but increased the respect in which they were held by the community.

He wanted a man who would take hold of one particular thing, and devote his entire time to it, and was willing to pay his share of the expense. Then, if they found anything they wanted analyzed, let them put it into the hands of their analytical chemist, and let him investigate it and give his opinion upon it. He would not say they should sanction that opinion, but let him give them the result of his knowledge and experience, and they could judge whether it was sound or not. There were a great many chemists scattered over the country, most of them good, but their investigations did not point directly to the dental profession. They wanted to make a dental profession mark in this business, and the only way to do it was in the manner proposed by the resolutions.

Dr. Newton, of Worcester, thought they needed information on subjects that went behind those matters which were commonly supposed to comprise the whole of their profession. He desired, particularly, to see the physiological department of dentistry investigated. Fathers and mothers often came to them and asked what they should do to give their children sound and healthy teeth. He confessed that he did not feel competent to counsel what should be done, and he feared many of his professional brethren were not. Until they learned these laws—laws of life as well as of science—they could not attain that high standard at which they all aimed.

Dr. Buckingham explained that in the remarks he had offered, he did not object to the appointment of the committee because he was opposed to the end sought to be gained, but because he thought it ought not to be brought into such a Convention as this. He had given as much money to advance his profession as his brethren, and he was willing to give, but he thought this was not the place for the introduction of the subject.

Dr. J. A. Perkins, of Rome, N. Y., said that he had long felt the need of something of the kind now proposed, and he hoped it would pass. If it did not bring in all it cost, he should be very much mistaken.

Dr. Fuller, of Portsmouth, N. H., desired to say a single word in favor of the resolution so ably advocated by Dr. Taft and other gentlemen. He would say, that although he was delighted to hear that Dr. Westcott had such a work in preparation, as had been alluded to, he still could not conceive of such a work, were it published to-day, as likely to benefit them in the investigations and discoveries that might take place before their next meeting, or in all coming time. He thought they could afford to employ a competent man, who had the ability to do the work, and he was in favor of starting the thing to-day, so that by next year they might have some report.

The question was then loudly called for, and being put, the resolution was declared adopted, there being but few dissenting voices.

Dr. Taft then offered the following resolution:

*Resolved*, That a Supervisory Committee of three from the profession in the United States, be appointed, whose duty it shall be to take charge of the whole matter, which committee shall have its Chairman, Secretary and Treasurer; and it shall also be the duty of said committee to secure the services of some competent person or persons, as experimenters, to commence their labors as soon as possible, and that the results of such labors shall be given to the profession as soon as obtained.

Drs. Allport, Taft, and Chandler, of Pennsylvania, spoke briefly in favor of the resolution. The last named gentleman said he was delighted with the proposition, for he thought it was the key by which they could arrive at the truth of certain questions which had been discussed in the Convention. He had become convinced that the destruction of the teeth was occasioned by the diseased condition of the saliva of the mouth; and by the analysis, by the experimenter it was proposed to appoint, of the alkalies and acids introduced into the mouth, they might arrive at the truth of the matter.

The question was then taken on the resolution, and it was adopted.

Dr. Taft then introduced the concluding resolution of his series, as follows:

*Resolved*, That such person be placed in the most favorable position, by the profession of the United States, for carrying out the objects of the previous resolution.

Dr. Taft said the object of the resolution was to pledge their influence and support to the gentleman who should be appointed to carry on these experiments, by aiding him in every way in their power. It was not contemplated that they should cease their experiments and investigations, but continue them as assiduously as possible, and give him such aid as they could.

The resolution was then adopted.

The President then announced the following gentlemen as the committee to prepare business for the next session of the Convention:

Drs. Jonathan Taft, Cincinnati, Ohio; Chapin A. Harris, Baltimore, Md.; C. W. Spaulding, St. Louis, Mo.; Benjamin Lord, New York City; Elisha Townsend, Philadelphia.

On motion of Dr. Buckingham, the thanks of the Convention were voted to the dentists of Boston, for the courtesies which had been extended to the members, and for the excursion down the harbor yesterday afternoon.

Dr. Clark, of New York, addressed the Convention on the subject of tempering instruments. He said that one of the greatest difficulties he had realized, in thirty-five years of practice, was that of securing instruments of the proper temper, for the various operations they were called upon to perform. The instrument makers declared that no two dentists could agree upon this subject, and they, therefore, made their instruments for those who seemed to require the greatest number. The great trouble was, that dentists were in the habit of having their instruments almost all of the same temper. He had been in the habit, for a great many years, of making instruments of different tempers, and he would state what tempers he thought best. Every one, he presumed, knew, that if a piece of steel was heated red hot, and plunged into any cold body, it would be perfectly hard. It must not, under any circumstances, be overheated. A cherry red, or a little lighter than that, would be sufficiently hot to produce hardness. That temper they wanted for their files and chisels, with which they intended to chip off the enamel, for their burrs, with which they intended to cut the enamel, and for their scrapers, which they used on the outside of the teeth. That was all, he believed, they required hard instruments for. For excavators or scalers, after brightening the instrument which has been hardened, the temper should be drawn by carefully heating it, until it assumed a tolerably bright straw color. This could be used for cutting bone, brass, gold, or soft iron, or anything except hard steel. In addition to that, they wanted instruments much softer, for strength. Pluggers required great strength—they did not want them to alter their shape. The greatest strength was found in the true spring temper, perhaps a little harder than was used for carriage springs. This was obtained in small instruments, by carefully drawing the temper beyond the straw color, till it became a blue, and a little dirty in its blue—changed a little toward white, as if it was burning off its color; then they had a strong spring temper. This, Dr. Clark said, was an important matter, for all dentists were obliged to temper their own instruments, as they could not rely upon the instrument makers; and in this matter, he found a greater variety of opinion than in any other. If the steel had been burned, they might throw it away, for it could not be restored.

Dr. Straw, of Bangor, said he was glad to hear the gentleman from New York speak of instruments, because it was a matter that ought to be understood. The method he adopted to secure the proper temper for *small* scrapers, was to heat them just right, and swing them in the air very rapidly. It was not necessary to draw the temper at all; this gave the best edge they could possibly have. It would not answer for *large* scrapers, however; they became too soft.

Dr. Randall, of Farmington, Maine, said that he was so situated, that he could not obtain instruments of a suitable temper, and had been compelled to manufacture them himself. After heating, as the gentleman from New York described, he plunged the instruments suddenly into a mercury bath, which produced the best temper.

Dr. Clark.—Is it not very hard?

Dr. Randall.—It is.

Dr. Clark.—Then it would be too hard for some purposes.

Dr. Priest, of Utica, remarked that a great deal of steel was spoiled by over-heating. To avoid this difficulty, instead of heating the steel with the lamp and blow-pipe, he heated a pair of tongs, and placed the small tools in them; in this way, there was no danger.

Dr. Clark.—I thank you for that information.

Dr. Lord, of New York, said he had found that one of the greatest difficulties in the way of success in dentistry, was the want of suitable instruments. The instrument makers did not understand their wants; the instruments they manufactured were those they *supposed* dentists wanted. He supposed a great many went to the instrument makers to procure their instruments, expecting them to be what they wanted; but they soon found their mistake, and that they must learn to make instruments for themselves, which he considered imperative for success. He thought one of the first things was to learn to make instruments, and adapt them to the peculiarities of a particular case. They did not need so many instruments, but they ought to be able to make them to hand, and they often were made thus, on an oil stone, or with a file, or with pliers, at any moment. He used an instrument with very little temper, for placing his foil; he wanted an instrument that would spring. He did not want it to bend, but he had rather it would bend and stay bent, than be rigid.

Dr. Straw said he had ascertained that it was necessary to hammer the point of an instrument before bending it—flattening the point upon two sides.

Dr. Miller said he wanted his light properly arranged, and then, with a good blow-pipe, he could do the work well. He did not heat the whole instrument red hot, only the cutting part. He heated it to a proper red and plunged it into soap or oil, and reduced it to a straw color. He could not buy such instruments.

Dr. Branch remarked that he learned from a watchmaker how to temper drills. He did it by holding a fine drill in the flame of a candle until it was properly heated, when he took it out and plunged it in the body of the candle. He used a sperm or stearine candle, which he thought preferable to a soft one. He believed he got a better cutting drill in that way than any other.

Dr. Clark said, that was the best temper for cutting steel, but he would not try it on brass.

Dr. Allport said that he was lately in the office of Dr. Rich, of New York, and he (Dr. R.) showed him his plan of bending instruments. He had adopted a system so that he always knew the exact curve of his instrument, and was never mistaken about it. Dr. Hawes, who was present, could explain the process better than he could, and he would ask him to do so.

Dr. Hawes said: In the first place they wanted an anvil on which to shape their instruments. Purchase a hammer and make an anvil of it by inserting it in a base. Draw the temper, and make some half dozen angles on the anvil, one at 20, 30, 40 or 50 degrees, as they wanted the angles of their instruments; then, with a die, number the angles, 1, 2, 3, and so on. Then, when that is complete, and the angles made perfect, and the anvil brought to a proper polish and finish, they would temper it again. Then, in order to shape their instruments to a proper angle, they had nothing to do but to lay them on the anvil, hit them one little tap with a hammer and the thing was done. Then take a die and mark the instruments No. 1, 2, 3, and so on, to correspond with the numbers on the anvil, and they knew just what instrument they took up, and if it was too straight or too much curved, they could instantly, by referring to the number, know the exact instrument necessary for their purpose.

Dr. Buckingham remarked that he had adopted a very simple process for drawing the temper of steel. He took a piece of gas pipe or an iron tube, say a quarter of an inch in diameter, filled it with charcoal, and put the point of the instrument in this, then laid it upon a piece of charcoal and heat to a red heat. There was no danger of injuring the steel in this way; it was almost as pliable as iron wire, and could be bent at will. To get the proper temper, he had used various substances, but common brown soap was as good as any thing. He heat the instrument up, put it into the soap, and heat up again, the soap would hold the heat, and then plunged it into some cold substances. This protected the steel from oxydation. With small instruments he put the point against a piece of steel, let the flame come up, and then, when he got the color he wanted, plunged it into water.

Dr. Clark said he had invariably found that if he heated his steel and cooled it, no matter how, it was always perfectly hard. He could not see any appreciable difference between water, oil or soap. It was sometimes troublesome to get instruments clean, but they could be cleaned in sulphuric acid. Early in life, before he thought of dentistry, he was told that if he wanted to keep a piece of steel handsome after it was hardened, he must envelop it in soap, which would clean off and leave the steel perfectly bright.

Dr. Leach said he was trained as a blacksmith, and he was proud of his early training. [Applause.] He had worked months and months on nothing but steel, and knew something about it. If they took a piece of steel, and made a slight mark across it with a coarse file, they took from its strength more than they would if they cut down the whole instrument to that size ; the whole spring was brought over one point. Hence it was important that every instrument should have as polished and smooth a surface as possible, and that the angle should not be too abruptly turned ; an angle turned with a perfect corner was not so strong as one that had a slight curve. In forming steel into instruments, as true a taper should be carried back from the cutting point into the socket or handle as possible, so that the strain will become gradual all along down to the point. If a shoulder was cut down to get a small size, the spring closed at that point, and a strain wrenched it off.

Dr. L. thought the best method of getting a spring temper had not been described. After getting the steel to a proper hardness, as they would for a cutting instrument, if they wished to get a low spring temper, put upon it common wax and hold it till that burns off, and the flame ceases. If a higher temper was desired, use sperm oil, which burns a little quicker; and if a still higher temper, sweet oil, and the moment the blaze ceases plunge it into water. In getting a spring temper, it was important that every part of the metal should be alike heated in drawing the temper, for if there was any one part more heated than another, the part not so much heated would break suddenly, because that would not yield while the other would.

Dr. Forbes, of St. Louis, said he had been convinced by the remarks on the subject of tempering steel, of the propriety of appointing an analytical chemist. Not a word had been said about the chemical properties of steel; why overheating steel destroyed it; what effect heating cast steel over a cherry red had upon it; why cast steel alone was destroyed by overheating, while German steel could be heated to a white heat, and plunged into water without being spoiled. There was only one method of properly tempering steel, if they all understood the effects of heat and cold. How was iron changed to steel? By carbon being incorporated into its pores. Why was steel made harder by heating and plunging it into oil, etc.? By the additional incorporation of carbon. The pores of the steel being opened by heat, the carbon is taken up, and the heat being suddenly checked, it was held there. Then, by opening the pores of the steel, and suffering the carbon to escape, they got just the proper temper. If this was well understood, they would all get just such instruments as they wanted.

Dr. Branch, of Illinois, then submitted the following resolution:

*Resolved*, That the Supervisory Committee, be requested to invite all members of the profession throughout the United States to participate in this movement, by contributing one dollar or more to the furtherance of the object.

Dr. Branch briefly advocated his resolution. This was a democratic convention, and the work was a democratic work, and he wished every member of the profession to have the opportunity of contributing to the object. He thought the day would come, when they would be proud of being engaged in this work, and posterity would bless them for it.

Dr. Kendrick, of Mississippi, submitted the following as a substitute for the resolution of Dr. Branch:

*Resolved*, That the Committee of Dental Investigation, be requested to invite all members of the Dental Profession in the United States, to contribute one dollar or more, together with any facts in their possession, calculated to further the objects of this movement.

Dr. Branch accepted the substitute, and it was adopted.

The President then announced the Supervisory Committee, as follows: Drs. Blakesley of Utica; Taft, of Cincinnati, and Buckingham, of Philadelphia.

A motion to adjourn *sine die* was then made but was not at once put.

Dr. Fuller, of Portsmouth, said he desired in a few words, to recommend the use of atmospheric plates for the insertion of a few teeth or a single tooth. He had employed them exclusively for two years, in every case where it was possible to do so, they gave the patient sufficient strength to masticate, and he was every way satisfied with the result.

Dr. Straw, of Bangor, gave an account of his manner of swaging plates. After cutting his male die, he imbedded it in sand, packing it down snugly as high as the labial edge; he then made his female die upon that; consequently, he got nothing but the central portion of the plate. He then put on his plate and struck it down, striking nothing but the centre, which goes down in two minutes. He then made another cast in the common way, and carried down the edges, previously, however, slitting the front part of the plate. He, also, if he found a prominence of the labial ridge, near the cuspids or bicuspids on either side, slit that, and carried the edges down; in two minutes more it was all done.

Dr. Ostrander explained an alcoholic lamp which he used, having a double tube, one of which is movable, to slide in or out. He slipped the slide inside the tube, covered it up, and the wick never clogged.

Dr. Fowler, of Yarmouth, Mass., explained a new blow-pipe, invented by Dr. Lawrence, of Lowell, which he said was exceedingly simple and very cheap.

Dr. Allen, of New York, expressed his regret that the subject of artificial dentistry had not been brought up. It was an important branch of the profession, and one well entitled to their consideration. He hoped that a place would be assigned for it in the discussions next year, and that it would not be set aside.

Dr. Coats of Virginia, explained his method of restoring gold. He had been in the habit of using the gold made by Jones, White & McCurdy, and it did not matter how far it had travelled, or how much it had been crumbled and knocked about, he brought it back to the same condition in which it left the hands of the maker. He had a little platina pan, about the size of a seidlitz powder box, or a sheet of gold foil, which was set in a frame over a spirit lamp, with sufficient heat to boil it full of water in a very few moments. Ascertaining what quantity of gold would be required, he took the requisite number of leaves and immersed them in this bath of boiling rain water, in which had been put about 40 drops of sulphuric acid. On removing them from the bath they would dry instantly. If it was examined by a microscope it would be found to have a pure and clean surface. They would find it work as well as any gold could. He dipped it into the solution in the rough condition. If examined carefully by the microscope before putting in the bath, it would be found that it had become filled with all sorts of foreign substances; and that was one reason why it was so difficult to make it adhesive. The annealing process did not take off all this foreign matter, but the bath did.

In answer to an inquiry, Dr. Coats said, that he put in only just so much as he intended to use at the time, for he held that the gold would receive impurities from the atmosphere. (Applause.)

Dr. Forbes offered the following resolution, which was unanimously adopted:

*Resolved*, That the thanks of this Convention be presented to the Reporters, for the able and impartial manner in which they have reported the proceedings of this Convention.

The Secretary *pro tem.* then read his minutes of the forenoon's proceedings, which were approved.

Dr. Wetherbee, of Boston, explained his method of inserting teeth upon healthy stumps. Instead of inserting the wooden pivot directly into the stump he filled part way down with gold, and inserted the pivot into that. In this way he preserved the stump in a healthy condition, as no moisture could possibly enter. Stumps treated in this manner would last for a great number of years. Sometimes he used a metallic cylinder, which was surrounded by gold, and thus avoided the use of wood entirely.

Dr. Forbes remarked that he was conscientiously opposed to pulling teeth. In some cases he forced a piece of gold wire down to the apex of the tooth, and left it there; then filled up the fang with fibrous asbestos, and the crown with gold. His operations in this way had been successful.

Dr. Newton recommended the use of nitrate of silver for cleaning out the fangs of teeth.

Dr. Miller stated, that on one occasion he was called upon to insert a plate in the mouth of a young girl 16 years old, and the mouth being very small, she was unable to retain it. The thought occurred to him that he would cut the frænum labiorum, and see what effect that would have. He raised the upper lip, took up a pair of scissors, and cut it the depth of a quarter of an inch. It was done very quickly. He found also some difficulty with the buccal muscles, and, raising the cheek, cut them in the same way. After the operation was performed, he placed the plate in her mouth, and it adhered quite firmly. By way of experiment he attached a weight to it, and found it would hold four pounds six ounces. He had performed this operation since, a number of times, and had never had any trouble. The incisions might bleed freely, but that could be stopped by the application of tannin or some other remedy. Dr. M., in reply to a question with regard to the safety of this operation, said he believed it was perfectly safe, but he should not resort to it unless obliged to.

Dr. Asay said he had found the same operation assist him very much indeed; but he thought it necessary to use a great deal of caution, and not cut too much. He had seen it have a very injurious effect upon a patient.

Dr. Miller remarked that he had never seen such a case.

The business of the Convention having been finished, the President, Prof. Taylor, rose, and spoke as follows:

GENTLEMEN.—We are now about to separate. We have had, at least it has seemed to me, very interesting and instructive sessions. But I really feel, and I think it is borne out by the discussions of this morning, that we do not allow members sufficient time for these annual convocations; that we come here unprepared in our arrangements to spend such a length of time as we find that the business of the Convention demands. Indeed, you have brought to my mind this morning, the old maxim, that we are *wedded* to our profession; and I have felt, whilst listening to the remarks that have been made, even since the motion was offered to adjourn *sine die*, that you would linger as the lover lingers with his sweetheart, after the parting kiss has been given, returning again and again. [Loud applause.]

My object in speaking at the present time, is, merely to say to you, that when we see you in the Queen of the West,—and I hope, gentlemen, (may that hope be realized!) that we shall see each and all of you, and not only you, but your friends and better-halves,—we shall give you a cordial greeting. Our city is large; the beautiful Ohio meanders close to our borders; we have every means for your accommodation and pleasure; and in behalf of my professional brethren of the West, I take this opportunity to say, we pledge you a hearty welcome. [Loud applause.]

The Convention then, at five minutes to 1 o'clock, P. M., adjourned *sine die*.

